# Maybe It's Not the Meth: Considering Biopsychosocial Contributors to Cognitive Impairment in Methamphetamine Polydrug Use

**DOI:** 10.3389/fpsyt.2022.795400

**Published:** 2022-02-14

**Authors:** James R. Gooden, Vanessa Petersen, Georgia L. Bolt, Ashlee Curtis, Victoria Manning, Catherine A. Cox, Dan I. Lubman, Shalini Arunogiri

**Affiliations:** ^1^Turning Point, Eastern Health, Richmond, VIC, Australia; ^2^The National Centre for Clinical Research on Emerging Drugs, University of New South Wales, Sydney, NSW, Australia; ^3^National Drug and Alcohol Research Centre, University of New South Wales, Sydney, NSW, Australia; ^4^Monash Addiction Research Centre, Eastern Health Clinical School, Monash University, Box Hill, VIC, Australia; ^5^Centre for Drug Use, Addictive and Anti-Social Behaviour Research, School of Psychology, Deakin University, Geelong, VIC, Australia

**Keywords:** methamphetamine, neuropsychology, addiction, acquired brain injury (ABI), drugs and alcohol, cognition

## Abstract

**Objective:**

In considering the cognitive harms of methamphetamine (MA) use, there is currently a limited appreciation of the profile of pre-existing, comorbid, or modifiable risk factors for cognitive impairment in individuals with MA-polydrug use who present to clinical services. This is in contrast to the well-recognized evidence in alcohol use groups. The aim of this study was to investigate the biopsychosocial and neuropsychological profiles of MA-polysubstance using individuals reporting cognitive impairment in comparison to an alcohol-using group.

**Methods:**

A retrospective file audit was undertaken of individuals who presented for assessment to a specialist addiction neuropsychology service and reported either more than 1 year of heavy MA use as part of a polydrug use history (*n* = 40) or having only used alcohol (*n* = 27). Clinical histories including demographic, medical, mental health, substance use, and neuropsychological assessment results were extracted from medical records. Between group comparisons were conducted to explore differences in the MA-polydrug vs. the alcohol group.

**Results:**

Individuals in the MA-polydrug group were significantly younger, commenced substance use at an earlier age, were more likely to have an offending history, and experienced an overdose than those in the alcohol group. No differences in comorbid neurodevelopmental, psychiatric or acquired brain injury diagnoses were observed between groups. For neuropsychological functioning, significant group differences were observed in overall IQ, semantic verbal fluency, and psychomotor tracking, where individuals in the alcohol group performed significantly worse.

**Conclusions:**

Neuropsychological profiles were largely equivalent between groups across cognitive domains, with minor differences in favor of the MA-polydrug group. Relative to the general population, cognitive functioning was reduced for both groups across a range of domains. High rates of comorbid mental health concerns were common across both groups, however, individuals in the MA-polydrug group presented with a higher risk of overall harm from substance use at a significantly younger age which is a unique concern for this group. These findings highlight the importance of considering the biopsychosocial factors, such as age of first use, emotional distress, indirect substance related harms including overdose and blood born virus infection that may be relevant to experiences of cognitive difficulty in MA-polydrug users.

## Introduction

Methamphetamine (MA), often colloquially known as ice or crystal meth, is a highly addictive derivative of amphetamine and has been increasing in illicit use within Australia (1). Between 1999 and 2017 a 4-fold increase in MA deaths was observed (2), with an estimated $5 billion societal cost relating to MA use in 2013 to 2014 (3). The acute effects of MA use include euphoria, increased alertness, hyper excitability, restlessness, and insomnia, while physiological effects include hypertension, vasoconstriction, and tachycardia (4). At the withdrawal stage, effects include dysphoria, depression, irritability, anxiety, poor concentration, hypersomnia, fatigue, paranoia, and craving (4). Collectively, these symptoms can have significant implications for everyday functioning in those with recurrent or dependent use. At a neurological level, MA acts on monoamine neurotransmitters including predominantly dopamine, and to a lesser extent serotonin and noradrenaline, which have widespread projections throughout the brain (5). As such, it has been proposed that MA can have neurotoxic effects via interacting mechanisms relating to hyperthermia, oxidative stress, toxic metabolites, neuroinflammation, and high cortisol levels (6). Therefore, a particular concern regarding the increasing prevalence of MA use is the impact that this may have on neuropsychological functioning in heavy or dependent users.

In practical terms, establishing the longer term neuropsychological effects of MA use is complicated by several clinical realities within the field of addiction. The first is that individuals with Alcohol and Other Drug (AOD) use disorders often present many years after commencing use, such as 18–20 years for alcohol (7), and consequently they rarely present with isolated issues. For instance, physical and psychiatric comorbidity is commonly observed in AOD cohorts (8). Secondly polysubstance use is considered the norm within these cohorts rather than the exception, with individuals often using multiple substances concurrently or having a history of using multiple substances over their lifetime (8, 9). As such, isolating the effects of one particular substance amongst others is inherently challenging.

These issues have been highlighted by several meta-analytic reviews which have attempted to elucidate the neuropsychological effects of MA use with mixed conclusions (10–12). The most recent of these identified small to moderate group deficits in several cognitive domains including learning efficiency, visual-spatial processing, comprehension, retrieval fluency, processing and psychomotor speed in abstinent MA users (10). Within each of these reviews is the clear observation that many of the included studies did not control for or report factors such as comorbid mental health conditions, premorbid IQ, alcohol or other substance use and duration of abstinence, all of which could have a confounding impact upon cognition (10, 11). Consideration of these broader contextual factors within research on individuals who use MA is critical given their high prevalence within substance using cohorts and the potential associations with cognitive functioning. For example, neurodevelopmental difficulties and reduced educational attainment are commonly observed within polysubstance using cohorts, which can account for aspects of cognitive functioning on assessment (13, 14). Similarly, emotional distress and psychiatric conditions have well established links with reduced cognitive functioning (15–17) and these conditions are frequently observed in substance using groups (18). In a recent study exploring the biopsychosocial predictors of neuropsychological functioning in a sample of treatment seeking substance users with cognitive concerns, factors including basic demographics, prescribed sedating medications, emotional distress, and formal diagnoses of acquired brain injury and neurodevelopmental conditions were found to have independent contributions to aspects of neuropsychological functioning (19). Despite the known impacts of broader contextual factors upon cognition in polysubstance groups more generally, there nonetheless continues to be a limited appreciation or recognition of the impact of pre-existing, comorbid, and/or modifiable risk factors for cognitive impairment in heavy MA-users.

In order to start addressing the limitations of prior work in MA cohorts, and proceed to identifying strategies that could be applied in clinical settings, the experiences of cognitive impairment in MA users (or more realistically described as “MA-polydrug users” from here on) and the context in which these difficulties occur need to be informed by real world clinical data. Work of this nature has already been conducted for alcohol use where there is a well-established evidence base for the long term deleterious effects heavy alcohol use on cognition (20, 21). These effects include persistent long term deficits in verbal learning, verbal memory, speed of processing and executive function (21), with chronic and extreme levels of use placing individuals at risk of sustaining alcohol related dementia (20). Furthermore, several key risk factors for sustaining cognitive impairment in alcohol using cohorts have been well-described, including older age, poorer general health and nutritional deficiency, chronic exposure and repeated withdrawal episodes (22–25). Crucially, awareness of these risk factors enables clinicians to implement appropriate harm reduction strategies where required. Given the availability of this evidence base and the known neurotoxic nature of alcohol, comparing the biopsychosocial profiles of MA-polydrug users to an alcohol only group would be beneficial in contextualizing the severity of any observed cognitive impairments and potentially inform treatment practices by identifying commonly occurring modifiable risk factors.

Therefore, the aim of this study was to conduct a comparison of the biopsychosocial and neuropsychological profiles of individuals with histories of heavy daily MA use as part of a broader substance use history (MA-polydrug group) and those reporting a history of only alcohol use (alcohol group). Based on existing literature, we predicted that individuals within the alcohol group would perform worse on all measures of cognitive functioning than individuals in the MA-polydrug group. While some degree of equivalence we expected between groups in terms of demographics and clinical comorbidities, this element of the study remained exploratory.

## Methods

### Setting

The Turning Point Addiction Neuropsychology Service is a government funded community-based service and is one of the specialist clinical services provided by Turning Point, a national addiction treatment and research center based in Melbourne, Australia. The clinic accepts referrals from community-based sources including corrections, drug and alcohol, mental health, general practice and case management services. Referrals range in complexity and include diagnostic queries in addition to assessments to support funding applications or inform treatment and care provision. To ensure referral appropriateness and service eligibility, all referrals are screened and triaged by clinical neuropsychologists. Further information regarding the service model has been previously described (14).

### Design

This study was a retrospective case file audit conducted following ethical approval from the Eastern Health Human Research Ethics Committee (Ref: LR88/2017).

### Participants

Participant data were extracted from an existing database of individuals seen for a neuropsychological assessment between August 2014 and December 2019. Only participants who had consented to having their data used for research purposes at the time of their neuropsychological assessment were included in the current study. Inclusion criteria for the service is that clients are aged over 18 and present with a significant past or current AOD history. Exclusion criteria for the service include referrals for decision making capacity or medico-legal purposes. For the MA-polydrug group, the database was reviewed to identify individuals who reported a lifetime history of at least 1 year of daily or near daily MA use in addition to providing valid test results on their neuropsychological assessment. Assessment validity was determined by the combination of clinical observation of test taking behavior and embedded measures of effort, with formal measures of validity being administered where there was an emerging suspicion of poor effort. With the overwhelming majority of individuals reporting daily use of at least one other substance, no exclusion criteria regarding alcohol or other substance use were set and so this group was defined as a MA-using polydrug group comprising 40 individuals.

For the alcohol group, the database was screened for individuals who met the following criteria i) reported a lifetime history of daily alcohol use and ii) reported no other significant illicit substance use histories (e.g., cannabis, amphetamines, or heroin) apart from once off or occasional experimental use, and iii) provided valid test results on their neuropsychological assessment. This yielded a final sample of 27 individuals.

### Measures

Data sources for the current study included information obtained from the comprehensive clinical histories taken during the assessment in addition to reviews of available medical records, neuropsychological test data, and clinical diagnoses, all of which were summarized in a client's neuropsychological assessment report. The following information was extracted: demographics including age, gender, years of formal education, offending history; medical history including: Hepatitis C status, the presence of a diagnosed Acquired Brain Injury (ABI) or neurodevelopmental conditions [e.g., intellectual disability, specific learning or language disabilities, and Attention Deficit Hyperactivity Disorder (ADHD)]; psychiatric comorbidities including histories of complex trauma (from childhood or adulthood), diagnosed conditions, suicidal ideation, and substance use histories. Medication sedative load was calculated using the Sedative Load Index (26–28), where medications are grouped according to their sedating properties and given a score of two (primary sedatives), one (sedation as an adverse effect or medications with a sedating component), or 0 (no sedating properties). A total sedative load score was derived by summing the rating scores for each medication prescribed to the individual.

#### Alcohol and Substance Use

Measures of alcohol and substance use included age of first use, years of use, and days of abstinence (i.e., difference between reported last substance use and day of assessment). Weekly alcohol use was recorded in terms of the number of standard drinks estimated to be consumed during a participant's heaviest period of alcohol use (29). This approach was taken to provide an indicator of the potential lifetime neurotoxic burden of alcohol use and risk of associated long term cognitive difficulty as opposed to recording recent use which may not be reflective of this risk (20). Sources for this data included available medical records and self-report during clinical interview at the time of assessment. In addition, for individuals in the MA polydrug group, details regarding their age of first use of MA, length of use, heaviest daily dose, and period of any abstinence was also recorded. Finally, current and lifetime frequencies of other substance use including cannabis, heroin, inhalants, GHB and hallucinogens were noted.

#### Neuropsychological Assessment

As part of assessment, a comprehensive neuropsychological battery was administered with measures being selected at the discretion of the treating neuropsychologist. Due to variability in the assessment batteries administered by clinicians, only the most consistently administered tests sampling the major cognitive domains were extracted from client records for the current study (Table 1). The most commonly administered measures included the Wechsler Adult Intelligence Scale Fourth Edition (31, 32) with prorated index scores for full scale IQ, verbal intellectual functioning, nonverbal intellectual functioning and information processing speed being utilized. The digit span subtest was selected as a measure of working memory in the absence of the working memory index being consistently available. Similarly, the logical memory subtest from the Wechsler Memory Scale Fourth Edition (31, 32) was utilized as a measure of verbal memory as this was frequently administered whereas other list learning tasks were utilized more interchangeably depending on clinical need. Other key assessment measures included the Rey Complex Figure Test (33) with the 30 min trial employed as a measure of visual memory, the Trail Making Test (39), Victorian Stroop test (38), Controlled Oral Word Association Test (38) and the Test of Premorbid Functioning (30). Emotional distress was measured using the Depression, Anxiety and Stress Scales (DASS) 21 Item version (37). All measures administered are routinely employed in clinical practice, well validated, reliable, and sensitive to changes in cognitive functioning as indicated by their inclusion in neuropsychological test compendia (38, 40). The following descriptors are used by our service to classify cognitive performances relative to normative data: Very Superior (98^th^ percentile and above); Superior (91^st^ to 97^th^ percentile); High Average (75^th^ to 90^th^ percentile); Average (25^th^ to 74^th^ percentile); Low Average (9^th^ to 24^th^ percentile); Borderline (2^nd^ to 8^th^ percentile); and Extremely Low (<2^nd^ percentile) (31, 32). For example, if a score falls at the 80^th^ percentile, the person has performed better on that task than 80 people out of 100. For further information on test interpretation and the relationships between standardized scores, percentiles and standard deviations refer to Strauss and colleagues (38).

**Table 1:** Neuropsychological assessment measures, variables used and normative data.

**Cognitive domain**	**Test**	**Variable used**	**Normative data**
Premorbid functioning	Advanced clinical solutions: test of premorbid functioning	Total score (scaled score)	Pearson Assessment (30)
Overall IQ	Wechsler adult intelligence scale: fourth edition	Full scale IQ (composite score)	Wechsler (31)
Verbal intellectual functioning	Wechsler adult intelligence scale: fourth edition	VCI index (composite score)	Wechsler (31)
Nonverbal intellectual functioning	Wechsler adult intelligence scale: fourth edition	PRI index (composite score)	Wechsler (31)
Working memory	Wechsler adult intelligence scale: fourth edition	Digit span (scaled score)	Wechsler (31)
Processing speed	Wechsler adult intelligence scale: fourth edition	PSI index (composite score)	Wechsler (31)
Verbal memory	Wechsler memory scale: fourth edition	Logical Memory II (scaled score)	Wechsler (32)
Visual memory	Rey complex figure test	30 min recall trial (z score)	Meyers (33)
Psychomotor tracking	Trail making test	Part A time taken (z score)	Tombaugh (34)
Divided attention	Trail making test	Part B time taken (z score)	Tombaugh (34)
Phonemic verbal fluency	Controlled oral word association test: FAS	Total words (z score)	Tombaugh (35)
Semantic verbal fluency	Controlled oral word association test: animals	Total words (z score)	Tombaugh (35)
Cognitive inhibition	Stroop test (Victoria version)	Color-word trial: time taken (scaled score)	Troyer (36)
Mood	Depression, anxiety and stress scale 21 item version	Total score for each subscale	Lovibond (37)

*Normative data for all measures were age corrected with the exception of the Trail Making Test which was age and education corrected. Further details for each test can be located through source publishers or reference texts as follows: Test of Premorbid Functioning (30); Wechsler Adult Intelligence Scale (WAIS-IV; (31)); Wechsler Memory Scale (WMS-IV; (32)); Trail Making Test (TMT; (39)); Rey Complex Figure test (RCFT; (33)); Stroop Test (38); Controlled Oral Word Association Test (COWAT; (38)); Depression, Anxiety and Stress Scale (DASS; (37)). PRI, Perceptual Reasoning Index; PSI, Processing Speed Index; VCI, Verbal Comprehension Index*.

### Procedure

All case files for individuals seen for assessment and who consented to their information being utilized for research during the audit period were reviewed by clinical neuropsychologists, de-identified, and relevant data including client histories, assessment results, and clinical diagnoses were extracted into a database. As per standard clinical practice, raw scores from neuropsychological assessment results were converted into scaled scores or z scores using age corrected normative data.

### Data Analysis

All variables met the assumption of normality, with the exception of age of first use, weekly alcohol use, and days of abstinence. For between group comparisons of categorical variables, Chi square tests of independence were used. Where the assumption of minimum cell sizes for Chi square tests was not met, Fischer's exact tests were utilized. For all continuous variables, independent measures *t*-tests were conducted to evaluate between group differences. For the three variables that did not meet the assumption of normality, Mann Whitney U tests were utilized as the nonparametric equivalent. Effect sizes (Cohen's d) were calculated using *t* and df values for between group measures. The alpha was set at 0.05 and all analyses, were two sided and conducted in SPSS Version 28 (41). A power analysis using G^*^power 3 for the main between group analysis indicated that an N of 52 was required to detect large effects with a power of 0.8 and alpha of 0.05 (42).

## Results

### Demographics

Participant characteristics and clinical comorbidity data for each group are presented in Table 2. At the time of assessment, individuals in the MA-polydrug group were significantly younger than individuals in the alcohol group, with a mean of 34 years compared to a mean of 49 years, respectively. The MA-polydrug group had a significantly higher proportion of males than the alcohol group. No significant differences in education were observed with both groups completing an average of 11 years of formal education. Education levels of Grade 10 or less formed the majority in both groups. A significantly higher proportion of individuals in the MA-polydrug group reported an offending history and within this, 42.5% were noted to have to a violent, as opposed to nonviolent, offending history.

**Table 2:** Participant characteristics and clinical comorbidity.

	**MA group**			**Alcohol group**			***df***	***t /* χ^2^*/ Z***	***p***	***d***
	***N (%)***	***M (SD)***	**Range**	***N (%)***	***M (SD)***	**Range**				
Demographics									
Age	40	34.35 (7.75)	20–61	27	49.44 (10.50)	26–66	65	−6.77	<0.001	−1.68
Gender (male)	31 (77.5)			14 (51.9)			1	4.81^c^	0.03	
Education years		11.13 (1.60)	8–16		11.50 (1.70)	8–14	64	−0.91	0.37	
< = Grade 8	5 (12.5)			3 (11.1)						
Grade 9–10	21 (52.5)			10 (37)						
Grade 11–12	10 (25)			7 (25.9)						
TAFE	3 (7.5)			5 (18.5)						
University	1 (2.5)			1 (3.7)						
Employment status									
Unemployed	26 (65)			11 (40.7)						
Employed/studying	6 (15)			5 (18.5)						
DSP/other	8 (20)			11 (40.7)						
Offending history	35 (87.5)			4 (14.8)			2	35.39^c^	<0.001	
Substance use									
Age of first use	39	14.13 (2.15)	8–18	22	18.68 (8.16)	11–48		−3.53^d^	<0.001	
Years of use	36	19.87 (7.72)	5–43	26	28.00 (11.85)	9–53	38.35	−3.11	0.004	−1.05
Days of abstinence	36	5.5 (121.63)^a^	0–2,190	23	7 (111.00)^a^	0–2,920		−0.29^d^	0.77	
Alcoholic drinks per week	39	86.70 (91.74)	0–294	27	175.41 (142.26)	31.50–588		−2.97^d^	0.003	
IV use	13 (32.5)			0						
Methamphetamine use										
Age of first use	32	22.94 (7.22)	12–40							
Days of abstinence	33	30 (188.00)^a^	0–2190							
Length of daily use										
1–5 years	18 (45)									
5–10 years	12 (30)									
10+ years	10 (25)									
Heaviest dose (grams/day)	25	1.20 (1.85)	0.1–7							
Mental health									
Diagnoses
None	12 (30)			8 (29.6)						
Depression	17 (42.5)			11 (40.7)						
Anxiety	13 (32.5)			12 (44.4)						
PTSD	5 (12.5)			2 (7.4)						
Bipolar	4 (10)			5 (18.5)						
Psychotic episodes	6 (15)			3 (11.1)						
Schizophrenia	0			2 (7.4)						
BPD	2 (5)			0						
OCD	1 (2.5)			2 (7.4)						
Other Diagnoses^b^	4 (10)			2 (7.4)						
Other PD	1 (2.5)			2 (7.4)						
Trauma history	15 (37.5)			13 (48.1)			1	0.61^c^	0.43	
Suicidal ideation							2	2.69^c^	0.26	
Past	12 (30)			13 (48.1)						
Active	4 (10)			1 (3.7)						
DASS scores										
Depression	33	18.85 (10.38)	4–38	18	15.03 (11.10)	0–36	49	1.23	0.23	
Anxiety	33	15.97 (9.37)	2–34	18	16.00 (9.38)	2–36	49	−0.01	0.99	
Stress	33	23.03 (9.07)	0–40	18	19.61 (11.99)	2–36	49	1.15	0.26	
Neurodevelopmental diagnoses									
None	31 (77.5)			22 (81.5)			1	0.16^c^	0.69	
ADHD	6 (15)			1 (3.7)						
Learning disability	3 (7.5)			3 (11.1)						
Language disability	0			1 (3.7)						
Intellectual disability	0			1 (3.7)						
Neurodevelopmental condition suspected but not formally diagnosed	4 (10)			3 (11.1)						
Medical									
Hepatitis C Status										
No history	30 (75)			26 (96.3)					0.009^e^	
Untreated	4 (10)			0						
Treated	5 (12.5)			0						
ABI risk factors										
None	16 (40)			12 (44.4)			1	0.13^c^	0.72	
Concussions or mTBI	17 (42.5)			5 (18.5)						
Moderate–severe TBI	2 (5)			2 (7.4)						
Non-traumatic ABI	5 (12.5)			8 (29.6)						
Overdose history	9 (22.5)			1 (3.7)					0.04^e^	
Medication sedative load	40	1.03 (1.76)	0–6	27	1.93 (1.47)	0–5	65	−2.19	0.03	−0.54

*ABI, Acquired Brain Injury; ADHD, Attention Deficit Hyperactivity Disorder; BPD, Borderline Personality Disorder; DASS, Depression, Anxiety and Stress Scales; DSP, Disability Support Pension; IV Use, Intravenous Use; mTBI, Mild Traumatic Brain Injury; OCD, Obsessive Compulsive Disorder; PD, Personality Disorder; TAFE, Technical and Further Education; TBI, Traumatic Brain Injury.*

a *Median and interquartile range.*

b *This includes Agoraphobia, Eating Disorders, Adjustment Disorder and Panic Disorder.*

c *Chi Square Statistic.*

d *Z Score for Mann Whitney U test.*

e *p-value from Fischer's Exact test*.

### Medical and Psychiatric Comorbidity

As expected, a high degree of formally diagnosed medical and psychiatric comorbidity was observed within both groups. Rates of formal mental health diagnoses were equivalent across groups overall, with depression and anxiety being the most common presenting conditions. For the MA-polydrug group this was followed by experiences of psychotic symptoms and post-traumatic stress disorder while in the alcohol group, bipolar and psychotic episodes were the next most common conditions. As shown in Table 2, in both groups, a high proportion endorsed a history of suicidal ideation and experiences of trauma with no differences between groups for either suicidal ideation or trauma. No significant differences were observed between groups in terms of self-reported experiences of depression, anxiety, and stress on the DASS. Of note, the mean reported scores for each domain of the DASS ranged between the moderate to severe ranges (e.g., 14–27 for depression and 10–19 for anxiety) for both groups indicating, on average, individuals were experiencing clinically significant symptoms of emotional distress at the time of their assessment.

No significant differences in the presence of a formally diagnosed neurodevelopmental condition (e.g., ADHD, learning, language or intellectual disability) was observed between groups, with ~20% in each group having a formal diagnosis. Of those without a formal diagnosis, a neurodevelopmental condition was strongly suspected, but not formally diagnosed, in 10% of cases in each group. Equivalent rates of ABI risk factors and diagnoses were observed between groups. Finally, a higher proportion of individuals in the MA-polydrug group reported past or current diagnosis of Hepatitis C, whereas individuals in the Alcohol group had significantly higher sedative medication loads.

### Substance Use

In comparison to the alcohol group, individuals in the MA-polydrug group reported a significantly earlier age of onset of substance use, with the MA-polydrug group commencing substances at a mean of 14 years of age compared to 19 years in the alcohol group. Consistent with the alcohol group being significantly older than the MA-polydrug group, individuals in the alcohol group reported significantly longer durations of substance use. Individuals in the alcohol group also reported significantly more alcohol consumed on a weekly basis during their heaviest period of use than the MA group. No differences in days of abstinence prior to neuropsychological assessment between groups were observed. In terms of risk of harm from substance use, as shown in Table 2, a significantly higher proportion of individuals in the MA group had overdosed, while no individuals in the alcohol group reported intravenous substance use in the past, compared to 32.5% of the MA-polydrug group.

For the MA-polydrug group, the average age of first use of methamphetamine was 23 years and 55% reported a history of use >5 years. The average daily amount consumed during the heaviest period of use was 1.2 grams a day and the median days of abstinence since last use was 30 days. Furthermore, in addition to MA use, individuals in the MA-polydrug group reported an extensive history of polysubstance use (Table 3). Most notably, just over half the group reported a history of daily or near daily alcohol and/or cannabis use. Furthermore, regular use of heroin, GHB, and ecstasy was also noted. Volatile inhalants, cocaine, hallucinogens, and ketamine use were less commonly reported. With regard to current use, 10% reported drinking on a daily or near daily basis, and 20% reported regular or near daily cannabis use around the time of their assessment. Finally, 7.5% reported maintaining a current pattern of heroin use.

**Table 3:** Proportion of clients in the Methamphetamine Group who used/use alcohol or other illicit substances on a regular or daily basis.

	**Past Use**		**Current Use**	

	**Regular %**	**Daily %**	**Regular %**	**Daily %**
Alcohol	22.5	55	22.5	10
Cannabis	12.5	57.5	12.5	7.5
Heroin	10	17.5	5	2.5
GHB	10	7.5	2.5	-
Ecstasy	20	5	-	-
Volatile inhalants	5	5	-	-
Cocaine	7.5	2.5	-	-
Hallucinogens	7.5	-	-	-
Ketamine	-	2.5	-	-

*Regular = More than monthly through to more than weekly. Daily = daily or near daily use. GHB, Gamma Hydroxybutyrate*.

### Neuropsychological Performance

The results of group performances on measures of neuropsychological functioning are presented in Table 4 along with the normal reference ranges indicative of where 50% of the general population would perform on these tasks based on normative data. As shown, for the MA-polydrug group mean performances in the domains of verbal intellectual functioning, nonverbal intellectual functioning, psychomotor tracking, and semantic verbal fluency were within the Average range (i.e., within the 25^th^ to 74^th^ percentile and −0.6–0.6 standard deviations away from the population mean). On the majority of the remaining measures, performances were below the normal reference range and fell in what would be classified as the Low Average range (i.e., 9^th^ to 24^th^ percentile and −1.3 to −0.6 standard deviations from the mean) relative to the general population. Furthermore, mean performances for visual memory and divided attention were well below the reference range falling with in the Borderline (i.e., 2^nd^ to 8^th^ percentile and −1.3 to −2.0 standard deviations from the mean) and Extremely Low ranges (i.e., <2^nd^ percentile and < -2.0 standard deviations from the mean). For the alcohol group, only the means for measures of verbal recall and semantic verbal fluency were within the normal reference range while mean performances for measures of psychomotor tracking and divided attention were within the Borderline and Extremely Low ranges. On all other domains, mean performances were within the Low Average range.

**Table 4:** Mean neuropsychological assessment scores^a^.

	**Normal reference range**	**MA group**			**Alcohol group**			***df***	***t***	***p***	***d***
		***n***	***M (SD)***	**Range**	***n***	**M (SD)**	**Range**				
TOPF	90–110	38	91.39 (12.03)	67–120	24	92.21 (14.22)	72–116	60	−0.24	0.81	−0.06
FSIQ	90–110	29	88.35 (10.65)	64–106	21	80.24 (11.45)	59–105	48	2.57	0.013	0.74
VCI index	90–110	36	90.17 (11.56)	70–122	25	84.08 (12.94)	61–110	59	1.93	0.06	0.50
PRI index	90–110	36	92.97 (11.90)	75–121	25	86.80 (13.87)	69–121	59	1.86	0.07	0.48
PSI index	90–110	37	87.32 (10.72)	56–108	24	83.00 (12.93)	62–108	59	1.42	0.16	0.37
Digit span	8–12	39	7.90 (2.06)	4–15	27	7.19 (2.32)	3–13	64	1.31	0.20	0.33
Logical memory	8–12	40	7.33 (3.02)	1–13	25	7.96 (2.65)	2–13	63	−0.86	0.39	−0.22
RCFT	−0.6–0.6	32	−1.53 (1.28)	−4.20–1.13	22	−1.14 (1.33)	−3.50–1.05	52	−1.09	0.28	−0.30
TMT-A	–0.6–0.6	37	–0.32 (1.10)	–3.07–1.31	23	–1.40 (2.32)	–6.77–1.07	58	2.43	0.018	0.64
TMT-B	−0.6–0.6	37	−2.25 (1.99)	−5.60–1.40	22	−2.44 (2.61)	−8.0–1.30	57	0.32	0.75	0.09
COWAT: FAS	−0.6–0.6	34	−0.65 (0.86)	−2.52–0.70	23	−0.63 (1.03)	−2.01–1.50	55	−0.05	0.96	−0.01
COWAT: animals	–0.6–0.6	34	0.40 (1.40)	–2.33–3.86	23	–0.53 (0.92)	–2.18–1.24	55	2.78	0.007	0.75
Stroop	8–12	33	8.18 (2.79)	0–15	26	7.73 (1.99)	4–11	57	0.70	0.49	0.19

*Refer to Table 1 for further detail on tests and variables used. COWAT, Controlled Oral Association Word Test; FSIQ, Full Scale Intelligence Quotient; PRI, Perceptual Reasoning Index; PSI, Processing Speed Index; RCFT, Rey Complex Figure Test; TMT-A, Trail Making Test Part A; TMT-B, Trail Making Test Part B; TOPF, Test of Premorbid Functioning; VCI, Verbal Comprehension Index.*

a *Measures were administered based on clinical judgement and so not all clients completed each measure*.

With regard to between group differences on standardized test scores, individuals in the MA-polydrug group performed significantly better than individuals in the alcohol group in the domains of overall IQ, psychomotor tracking, and semantic verbal fluency with medium effect sizes observed (Table 4). No other significant differences between groups were observed.

## Discussion

The aim of this study was to investigate the cognitive and biopsychosocial profiles of a group of individuals with a history of heavy MA use who presented to our specialist addiction neuropsychology service with cognitive concerns in comparison to individuals who reported only using alcohol. Overall, while some significant between group differences were observed in basic demographic variables, substance use, and aspects of cognitive functioning, the majority of presentations were consistent across groups, particularly in terms of clinical comorbidity and diagnoses. Our hypothesis that individuals in the alcohol group would perform worse in all cognitive domains was only partially supported, with individuals in the alcohol group having significantly lower full scale IQ scores, and worse performances on measures of psychomotor tracking and semantic verbal fluency, while no significant differences were observed in the remaining cognitive domains.

Of note, both the alcohol and MA-polydrug groups performed below normative reference ranges across a variety of cognitive domains, highlighting the broad nature of the difficulties experienced by these individuals. Such reductions can have significant functional implications for treatment engagement (43–45), as well as broader life participation. The finding that individuals in the alcohol group exhibited significantly lower overall full scale IQ scores (despite no differences being observed on a measure of estimated premorbid functioning) may reflect the generalized reductions in cognition that could be expected following extended periods of heavy daily alcohol use (21, 25). Being significantly older, individuals in this group also presented with significantly longer durations of substance use which may also account for differences in this overall domain as both age and lifetime histories of alcohol dependence have been associated with increased risk of cognitive impairment (25). Significantly slower psychomotor tracking performances, as measured by the Trail Making Test Part A (TMT-A), were also noted for the alcohol group in comparison to the MA-polydrug group. This is in accord with previous literature that has shown heavy drinking can have deleterious effects upon performance on this task (46). The difference in psychomotor tracking performances between groups may also be related to the significantly higher medication sedative loads in the alcohol group relative to the MA-polydrug group, which may have slowed the speed of their responding more generally (47). Indeed, other speeded tasks (as per the Processing Speed Index) for the alcohol group tended to fall below same-aged peers, in the Borderline range, and whilst the difference did not reach significance between groups, this does raise the possibility that slower speed in part contributed to poorer performance on TMT-A for these individuals (46). Of interest, both groups performed equally poorly on the second part of this task (Trail Making Test-Part B, TMT-B) which includes executive and attentional switching components in addition to the speeded elements of the first part (38). When considering performances on TMT-B relative to TMT-A for both groups, these findings might suggest that those in the MA-polydrug group in particular were having more difficulty with specific aspects of executive functioning and attentional switching (over and above psychomotor tracking), whereas the alcohol cohort had more generalized reductions across domains. Previous research has highlighted reduced performances in these higher order cognitive domains of executive functioning and attention in MA-polydrug users (10–12). Consistent with our previous work (14), the Trail Making Test was the most sensitive to eliciting cognitive impairments with mean scores in both groups being over two standard deviations below normative data.

Despite being significantly younger than the alcohol group, the MA-polydrug group presented with largely similar cognitive profiles and comorbidities. This is of clinical concern, as it is not clear whether this places MA-polydrug users at risk of experiencing an accelerated trajectory of cognitive impairments due to cumulative factors over time, with potential for poorer overall outcomes at an earlier age than other groups. With individuals in the MA-polydrug group, on average, commencing substance use at a significantly earlier age and across a critical period of neurodevelopmental maturation, this may further increase their risk of poorer neurological, psychiatric, and psychosocial outcome (48). In order to reduce risk of adverse long term effects in this MA-polydrug group, access to early intervention and supports is critical.

With regard to biopsychosocial factors, the most immediate and striking characteristic of the MA-polydrug group is that they are best described as a polydrug group considering the high rates of alcohol, cannabis, and other substance use noted in their histories and the findings of this study should be interpreted in that context. In order to obtain the most ecologically valid sample of MA users for this study, the only criteria for the MA-polydrug group were in relation to having a lifetime history of daily MA use for over 12 months. With the overwhelming majority reporting use of other substances apart from MA, the current sample reflects the well-established clinical perspective that polysubstance use among these cohorts is the norm rather than the exception (9). This has significance from a neuropsychological perspective as the extent of polysubstance use should not be underestimated, particularly when interpreting cognitive test performances. For instance, as would be expected, individuals in the alcohol group consumed a significantly higher amount of alcohol per week during their self-reported lifetime heaviest period of use. However, the mean weekly units of alcohol consumed by individuals in the MA-polydrug group during their heaviest period of use was still well in excess of recommended guidelines (49), with over half reporting a history of daily drinking. Furthermore, the mean weekly consumption was equivalent to 12 standard drinks a day and sustained use at this level over many years may result in declines in cognitive functioning and could partially account for some of the difficulties experienced by individuals in this group (21). Thus, these findings highlight the importance of considering the broad history of AOD use rather than simply focusing on the most recent, current, or principal drug of concern when considering the possible etiologies of cognitive difficulty.

In addition, those in the MA-polydrug group presented with significantly greater substance-related harms relative to the alcohol only group. As would be expected from a polydrug group, a younger average age of onset of use, increased risk of overdose, use of intravenous methods, and blood borne virus infection were present. The MA-polydrug group also presented with significantly higher rates of offending histories with a high proportion having a history of violent offending (42.5%) which is consistent with prior studies (50, 51). Interestingly, while no differences were observed in diagnoses of moderate or severe ABI, a high proportion of individuals in the MA-polydrug group (42.5%) reported having sustained concussive or mild traumatic brain injuries. This may be reflective of the higher rates of offending behavior and/or potential violent interactions experienced by individuals over the years associated with a substance using lifestyle (50). The differences in overall offending and legal involvement also suggests that individuals in the MA-polydrug group may be more likely to come to the attention of authorities, potentially through their involvement in illicit as opposed to licit substance use (i.e., alcohol). Past research has demonstrated the increased risk of legal involvement, incarceration, and adverse psychosocial experiences of individuals with MA use disorder relative to other substance using groups (8, 9). This may also be one reason why individuals in the MA-polydrug group were significantly younger than those in the alcohol group; with involvement in the legal system they, and their experience of cognitive difficulty, may come to the attention of clinical services and be referred earlier than other groups. Similarly, the use of illicit substances by this group may result in increased formal and informal societal pressure for individuals to seek treatment earlier than those using a licit and socially sanctioned substance such as alcohol (8).

The high prevalence of mental health comorbidity, trauma experiences, suicidal ideation, and active symptoms of depression, anxiety, and stress in both groups highlights the common experience of emotional distress in these substance using cohorts. While these findings are consistent with prior research in substance using cohorts (8, 9), with some associations between emotional distress and intellectual functioning being demonstrated (52), the significant impact of emotional distress upon cognitive functioning is often not appropriately considered. Such symptoms are critical to consider as the presence of mental health and psychiatric comorbidities represent potentially modifiable risk factors for cognitive impairment (15–17, 19, 53). For example, in a recent study of the biopsychosocial predictors of neuropsychological functioning in substance users attending for neuropsychological assessment, emotional distress was consistently shown to have an independent contribution to test performances on several cognitive domains including information processing speed, working memory and divided attention (19).

Similarly, premorbid characteristics such as pre-existing neurodevelopmental disabilities were also common and could account for some aspects of cognitive difficulty in these cohorts (13, 19, 54). It was notable that 15% of the MA-polydrug cohort had diagnoses of ADHD, which is higher than rates in the adult population more broadly and in keeping with previous literature that has suggested rates of ADHD are between 2 and 6 times higher in MA users (55, 56). These neurodevelopmental and substance-related factors may well have an interconnected bidirectional relationship, whereby pre-existing cognitive difficulties (i.e., ADHD) may increase likelihood of risk-taking behavior at a young age, including MA use and associated harms, but MA use may also be an indirect way that this group “self-medicates” for ADHD symptomatology (55). From a clinical perspective, these finding highlight the need to consider neurodevelopmental disorders (particularly ADHD) and mental health diagnoses and implement appropriate intervention as part of the treatment process for this group.

Overall, the implications of these findings are that a wide variety of risk factors and clinical variables need to be considered when evaluating the presence of cognitive impairment in people who use substances including medical and psychiatric health, active and past substance use, emotional distress, and psychosocial background including adverse childhood events and educational opportunities. Literature that makes strong statements about the presence of cognitive impairments being solely attributable to MA use, without considering the implications of these broader factors should be interpreted with caution. The contribution of biopsychosocial factors to cognitive functioning has been well established (15–17, 19, 53, 57–63) and should not be underestimated in individuals using MA or other substances who present with cognitive difficulty. It is also clear that individuals with MA-polydrug use histories and cognitive difficulty present with some unique aspects to their biopsychosocial profiles including exposure to a higher risk of overall harm from substance use at a significantly younger age.

From a clinical perspective, these findings reiterate the need for adopting a biopsychosocial approach to formulation and ensuring that all needs are identified and addressed in order to maximize overall outcomes for MA-polydrug users. Those querying MA or substance-related cognitive impairment should consider the impact of these other, potentially modifiable factors that are known to impact cognition, prior to referral to neuropsychology or conveying a diagnosis of ABI. For example, in collaboration with the individuals' goals this many include referrals to review any medical or psychiatric concerns, trialing participation in drug and alcohol rehabilitation or addressing unmet psychosocial needs (e.g., housing or legal issues). Addressing these may allow for other interventions or treatments to be more effective (e.g., neuropsychological assessment, psychological therapy or counseling). Should an individual then experience persistent cognitive difficulty or a failure to recover adequately this would be a particularly clear indication for the need for further neuropsychological input or investigation. Recognizing that achieving the above is often out of reach when seeing individuals with complex and ongoing use, in these circumstances, neuropsychologists should also be cautious when attributing cognitive impairments solely to MA use. Rather, clinicians need to consider broader substance use histories, the indirect impact of associated substance-related harms, and the potential for co-morbid neurodevelopmental disorders, mental health and other psychosocial/legal stressors. Neuropsychological interventions should also be holistic in their approach, addressing cognitive, psychological, emotional, behavioral, and lifestyle factors. From a research perspective, the current findings also highlight the limitations of grouping individuals according to their substance use as this is fraught with issues and potential confounding variables for all but the most rigorous experimental designs. An alternative approach may be to adopt more transdiagnostic methods to clinical classification given the breadth of common comorbidities and symptomatology across substance using cohorts. A particular avenue for future research would be to evaluate the independent contributions of these demographics, clinical and substance use variables on cognitive functioning in particular groups such as individuals who use MA heavily which would help inform clinical formulations and treatment recommendations. Longitudinal follow up of a cohort such as this would be particularly informative.

## Limitations

The findings of the current study must be interpreted in the context of a number of limitations. Firstly, the sample was drawn from existing data of clients seen for a neuropsychological assessment, which limited what variables could be extracted and utilized in the analyses. Furthermore, as assessments were conducted for clinical, rather than research purposes, not all clients completed the same measures leading to some missing data throughout several variables. In order to address this limitation, the most consistently administered measures were selected for the study to maximize the data available. Another limitation was the smaller sample size of the alcohol group which likely limited our ability to detect differences with small effect sizes and conduct more advanced statistical procedures such as multiple regression to evaluate the predictive utility of key variables. Also as noted, the MA-polydrug group consisted of individuals who also used a wide variety of other substances, including alcohol, which may have impacted our ability to detect differences between the two groups. Consideration of methods to control for these effects at a statistical level was given, however, due to the concerns regarding the appropriateness of these methods with the current sample, power and available variables we elected not to pursue these approaches. Similarly, consideration was given to adjusting for multiple comparisons, such as using Bonferroni corrections, however this would have resulted in an overly strict alpha level (64). Finally, these findings must be interpreted within the context of the clinical setting that the participating individuals were referred to which is a tertiary specialist clinical neuropsychology service. As part of this service individuals are triaged according to clinical complexity and need, with those experiencing more persistent or significant cognitive difficulties more likely to be recommended to undergo a formal assessment. Individuals presenting with less significant concerns in combination with unmanaged treatment needs are frequently recommended to pursue these treatment avenues in the first instance prior to formal assessment. This clinical triage process therefore likely introduces a degree of selection bias for the sample obtained and may not be representative of wider clinical populations.

## Conclusions

The findings of the current study highlight the clinical comorbidities and biopsychosocial contexts of individuals with histories of heavy MA use who are experiencing cognitive difficulty. In these contexts, it is often not possible to attribute causality or identify MA use as a specific etiology, as many of these other demographic, neurodevelopmental, medical, psychiatric or polysubstance use factors are well known to significantly influence cognitive functioning. Importantly, these experiences of cognitive difficulty were sufficient to warrant formal neuropsychological investigation and consistent with prior work, a range of modifiable and non-modifiable risk factors for cognitive decline were present, such as heightened emotional distress, that may account for these difficulties (19). These factors have important implications for the interpretation of past and future research work and also for clinicians working with these cohorts as they must be well attuned to the presence of these factors and able to ensure they are well managed through appropriate referral and treatment.

## Data Availability Statement

The data analyzed in this study is subject to the following licenses/restrictions: the dataset analyzed is not publicly available in order to maintain confidentiality of individual client data but may be available from the corresponding author on reasonable request with appropriate organizational and ethical approval. Requests to access these datasets should be directed to jamesg@turningpoint.org.au.

## Ethics Statement

The studies involving human participants were reviewed and approved by Eastern Health Human Research Ethics Committee, Eastern Health, Box Hill, Australia. The patients/participants provided their written informed consent to participate in this study.

## Author Contributions

JG, CC, VP, SA, and VM: design and conduct of the study. JG, CC, VP, and AC: data collection and extraction. JG, VP, and SA: data analysis. JG, VP, CC, SA, GB, AC, VM, and DL: manuscript drafting, editing and review. All authors have read and approved of the final manuscript.

## Funding

JG is supported by a scholarship from the National Centre for Clinical Research in Emerging Drugs (NCCRED), funded by the Commonwealth Department of Health (Australia). NCCRED had no role in the review, design, analysis, interpretation or preparation of this work.

## Conflict of Interest

DL has received travel support and speaker honoraria from Astra Zeneca, Bristol Myers Squibb, Camurus, Indivior, Janssen, Lundbeck, Servier, and Shire. The remaining authors declare that the research was conducted in the absence of any commercial or financial relationships that could be construed as a potential conflict of interest.

## Publisher's Note

All claims expressed in this article are solely those of the authors and do not necessarily represent those of their affiliated organizations, or those of the publisher, the editors and the reviewers. Any product that may be evaluated in this article, or claim that may be made by its manufacturer, is not guaranteed or endorsed by the publisher.
